# Effect of Air Pollution on Obesity in Children: A Systematic Review and Meta-Analysis

**DOI:** 10.3390/children8050327

**Published:** 2021-04-23

**Authors:** Nichapa Parasin, Teerachai Amnuaylojaroen, Surasak Saokaew

**Affiliations:** 1 School of Allied Health Science, University of Phayao, Muang 56000, Phayao, Thailand; 2 Atmospheric Pollution and Climate Change Research Units, School of Energy and Environment, University of Phayao, Muang 56000, Phayao, Thailand; teerachai.am@up.ac.th; 3 Division of Pharmacy Practice, Department of Pharmaceutical Care, School of Pharmaceutical Sciences, University of Phayao, Muang 56000, Phayao, Thailand; surasak.sa@up.ac.th; 4 Unit of Excellence on Clinical Outcomes Research and Integration (UNICORN), School of Pharmaceutical Sciences, University of Phayao, Muang 56000, Phayao, Thailand; 5 Center of Health Outcomes Research and Therapeutic Safety (Cohorts), School of Pharmaceutical Sciences, University of Phayao, Muang 56000, Phayao, Thailand

**Keywords:** air pollution, children, obesity

## Abstract

Air pollution exposure has been identified as being associated with childhood obesity. Nevertheless, strong evidence of such an association is still lacking. To analyze whether air pollution exposure affects childhood obesity, we conducted a systematic review and meta-analysis utilizing the PRISMA guidelines. Of 7343 studies identified, eight studies that investigated the effects of air pollutant characteristics, including PM_2.5_, PM_10_, PM_coarse_, PM_absorbance_, NO_x_, and NO_2_, on childhood obesity were included. The polled effects showed that air pollution is correlated with a substantially increased risk of childhood obesity. PM_2.5_ was found to be associated with a significantly increased risk (6%) of childhood obesity (OR 1.06, 95% CI 1.02–1.10, *p* = 0.003). In addition, PM_10_, PM_2.5absorbance_, and NO_2_ appeared to significantly increase the risk of obesity in children (OR 1.07, 95% CI 1.04–1.10, *p* < 0.00; OR 1.23, 95% CI 1.06–1.43, *p* = 0.07; and OR 1.10, 95% CI 1.04–1.16, *p* < 0.001, respectively). PM_coarse_ and NO_x_ also showed trends towards being associated with an increased risk of childhood obesity (OR 1.07, 95% CI 0.95–1.20, *p* = 0.291, and OR 1.00, 95% CI 0.99–1.02, *p* = 0.571, respectively). Strong evidence was found to support the theory that air pollution exposure is one of the factors that increases the risk of childhood obesity.

## 1. Introduction

Over the last few decades, the number of people suffering from obesity has risen at an unprecedented rate around the world. For example, in 2016, nearly 40% of men and 15% of women worldwide were overweight, with 11% of men and 15% of women being obese [1]. Obesity is estimated to have contributed to over 35.8 million adjusted livelihoods and 2.8 million deaths worldwide as well as representing 2.8% of the global GDP in 2014. Obesity and being overweight are major risk factors for a variety of chronic diseases, including diabetes, cardiovascular disease, kidney disease, and cancer. In response to the obesity epidemic, numerous studies have been conducted to better understand the complex and varied causes of obesity. In the study by Kim et al., it was unexpectedly discovered that air pollution can cause obesity in young adults by slowing their metabolism [2]. This finding corresponds to the hypothesis that 90% of the world’s population now lives in areas with poor air quality. As a result, policymakers must gain a better understanding of the link between air pollution and obesity.

According to the air pollution hypothesis, there is a connection between air pollution and weight gain through biological and behavioral mechanisms. According to Kim et al., the major pollutants causing obesity are nitrous oxides (NO_x_), nitrogen dioxide (NO_2_), ozone (O_3_), and particulate matter (PM_10_ and PM_2.5_). Higher levels of long-term NO_2_ exposure are associated with a low-density lipoprotein (LDL)-cholesterol levels of 11.4 or 9.4 mg/dL [2]. Additionally, [3] reported that a similar group of air pollutants, namely, PM, NO_2_, SO_2_, and O_3_, have significant impacts on obesity [3]. Focusing on children, obesity is emerging as a multifactorial disease due to interactions among genetics, nutrition, and lifestyle [4]. The most critical factor is differences in nutritional intake. In the last decade, there has been growing concern that environmental chemicals might contribute to higher rates of obesity [5]. Epidemiological research has correlated the body mass index (BMI) with environmental contaminants such as nitrogen oxides (NO_x_), nitrogen dioxide (NO_2_), PM_2.5_, PM_10_, and PM_coarse_, which are components of city air pollution [6,7]. Recent research in the United States showed that exposing teenagers to levels of NO_x_ emissions was positively correlated with abnormal or stunted heights in children aged up to 10 years [8,9,10]. However, Alderete et al. [8] and Mao et al. [11] have shown inconclusive findings regarding the association between PM exposure during pregnancy and the rate of childhood obesity. In cohort studies from the United States, low concentrations of PM_2.5_ in children were found to be related to childhood obesity and BMI in adolescence.

Even though many previous research studies have shown the impact of air pollution on weight status, there have been some differences between these studies. For example, in the study by An et al. [3], it is reported that 44% of previous studies found a positive connection between air pollution and body weight status, also 44% of the recently studies found a null finding, while the remaining 12% found no statistically significant correlation. The latest evidence regarding the connection between air pollution and obesity is ambiguous, especially in children. For example, Nikolic et al. [12] found that air pollution negatively contributed to children, whereas Jerrett et al. [13] reported that the exposure to air pollution causes significant effect on BMI level in children. To address this ambiguity, we performed a systematic review and meta-analysis in this study to investigate the relationship between air pollution exposure and childhood obesity.

## 2. Materials and Methods

This systematic review and meta-analysis were carried out in compliance with the application submitted to the Preferred Reporting Items for Systematic Reviews and Meta-Analysis Statement (PRISMA) [14]. This research has been registered via PROSPERO (CRD42020214290 registration number).

### 2.1. Data Sources and Search Strategy

Three databases, PubMed, Science Direct, and Cochrane Library, were systematically searched for data between the years 2010 and 2020. The last search was conducted on 30 September 2020. The keywords related to the medical subject were applied as appropriate. The publications’ bibliographies were researched for related articles. A search strategy was implemented using the following keywords: “Air pollution” or “PM_10_” or “PM_2.5_” or “dust” or “fine particle” and “overweight” or “weight” or “BMI” and “child” for all databases. There were no restrictions on the design and language of the study.

### 2.2. Study Selection

The inclusion criteria were as follows: (1) the study of children aged 0–19 years who were exposed to air pollution; (2) the study measured the Body Mass Index (BMI), (3) the study provided the odds ratio (OR), risk ratio (RR), hazard ratio (HR), 95% confidence interval (95% CI), or *p*-value, and (4) the study published between year 2010 and 2020. Studies on animals were not presented as original research, e.g., reviews, comments, editorials, opinions of experts, inquiries, letters, abstract conferences, case reports, systematic reviews, meta-analyses, and series, were excluded. Studies that did not include impact estimates or failed to provide enough data to calculate impact estimates have also been eliminated.

### 2.3. Outcome Measures

For the purposes of this study, “results” refers to the Body Mass Index (BMI) of the child. The weight and height of each participant were measured using a clinical standard protocol. There are several criteria that refer to overweight or obesity that are used in various regions but in this paper, the definitions of “overweight” and “obesity” used were as follows:

(1) The World Health Organization (WHO) 2007 Growth Charts: overweight is a z-score of > + 1 (equivalent to BMI 25 kg/m^2^) and obesity is a z-score of > + 2 (equivalent to a BMI equal to 30 kg/m^2^) [15].
(2) The Centers for Disease Control and Prevention (CDC) BMI growth charts: normal weight ≤ 85th percentile, overweight = 85th to 95th percentile, and obesity ≥ 95th percentile [16].
(3) Body Mass Index (BMI) cutoffs by age and sex in accordance with the International Obesity Task Force (IOTF) at the age of 18 [17].
(4) The Chinese national standard Screening for Overweight and Obesity among School-age Children and Adolescents calculated using BMI and cutoff by sex and age group [18].
(5) The national Obesity Observatory and the population cut-off taken as greater than the 85th percentile [19].

The term “exposure to air pollution” refers to childhood exposure (at ages 0–19 years), including indoor and outdoor air pollution.

### 2.4. Data Extraction and Quality Assessment

Each title, abstract, and full-text article was individually reviewed by two researchers (N.P. and S.S.) to identify potentially qualifying studies, and discrepancies were addressed through discussions with a third researcher (T.A.). The same researchers carried out data extractions from all potentially relevant articles. Each study contained the keywords “air pollution,” “obese,” “child overweight,” “BMI,” intervention/exposure details, results in detail, effect size (such as OR, RR, or HR, etc.), and 95% CI or standard errors. The following information was extracted from each study: authors, publication year, country, study design, study population, percentage of males, pollutant tested (average exposure), exposure assessment method, duration of follow-up, BMI, statistical model, adjusted covariates, number of overweight and effect size (such as OR, RR, or HR), and their 95% CI or standard error values. When the results were missing, the authors of the study were contacted, and studies were excluded if the authors did not reply within 1 month. Two researchers (S.S. and N.P.) independently investigated all extracted data.

Two researchers (S.S. and N.P.) independently evaluated the quality of each study. The Risk of Bias (RoB) tool for randomized controlled trials of the Cochrane Collaboration was used. The Newcastle-Ottawa Scale (NOS) was the analytical technique used to assess observational studies [20]. The NOS allocates a maximum of nine points, with a total of >7 high-quality studies. Determination of which Downs and Black studies to include and resolution of discrepancies were done through discussion.

### 2.5. Data Synthesis and Statistical Analysis

In order to determine the association between air pollution exposure and child obesity, the DerSimonian–Laird random-effects model was used to calculate overall ORs and 95% CIs. If the adjusted OR was reported, the adjusted OR was selected. The heterogeneity of the Cochran Q statistics was also investigated. For each analysis, an alpha value of 0.10 was selected to indicate heterogeneity in trials. The heterogeneity degree with I^2^ values has been shown. I2 values above 75%, from 75% to 25%, and below 25% indicate high, moderate, and low levels of heterogeneity, respectively [21]. In the case of heterogeneity, an attempt to explore potential heterogeneity sources was made. Bias in publishing was evaluated using Egger’s test and the funnel plot [7,22,23]. Publication bias was indicated at *p*-value < 0.05 [21]. The trim-and-fill method was also applied for calibration of the publication bias.

### 2.6. Sensitivity and Subgroup Analysis

For the evaluation of our analysis, a sensitivity analysis and subgroup analysis were carried out using a pooling model (random effect vs. fixed effect), the area of exposure, and the duration of exposure. Unadjusted OR articles were omitted from our analysis.

## 3. Results

### 3.1. General Information

**Figure 1:**
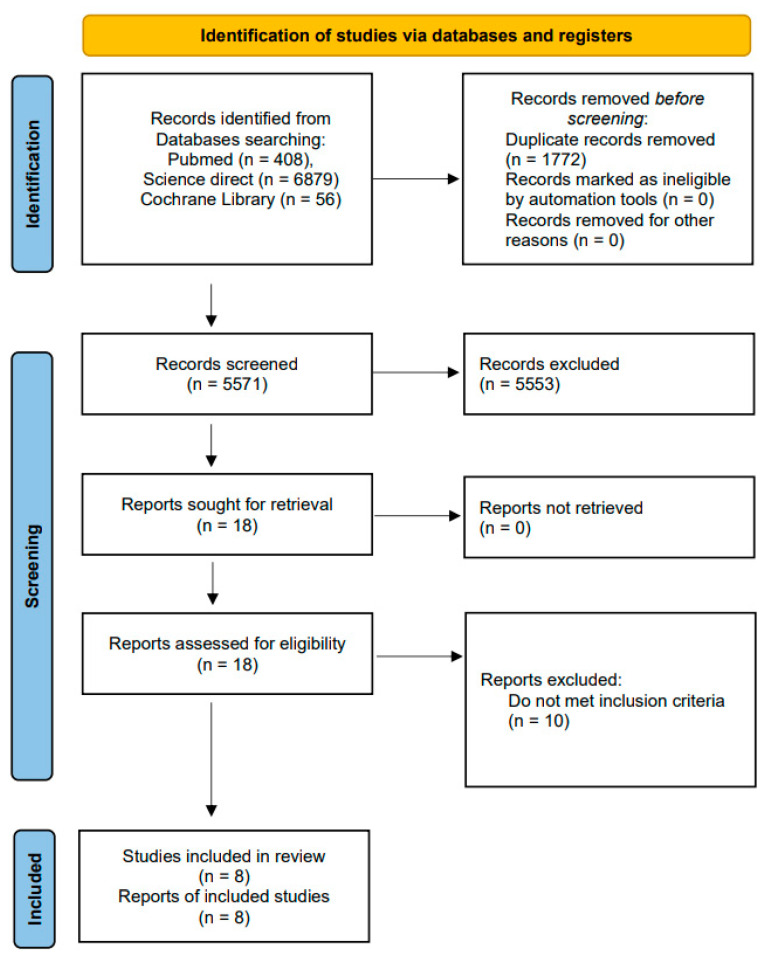
Flow diagram of the studies identified; eight studies were selected to study the effects of air pollutant on childhood obesity [24].

The literature search process is displayed in Figure 1. The literature search identified 7343 papers (408 results from PubMed, 6879 results from Science Direct, and 56 results from Cochrane Library). Duplicates accounted for 1772 articles, and these were eliminated. After screening the titles and abstracts, 5773 were excluded for a variety of reasons (Figure 1). Finally, the meta-analysis was carried out on eight eligible original articles with a total of 101,022 participants. Table 1 summarizes the measures of air pollution and body weight status and the statistical models adopted by the selected studies. The most commonly examined air pollutants included PM_10_, between 2.5 and 10 µm (particulate matter with a diameter between 2.5 and 10 µm), PM_coarse_, PM_2.5_, PM_2.5absorbance_ (a marker of black carbon), nitrogen dioxide (NO_2_), and nitrogen oxides (NO_x_). The primary aspects of the eight articles are mentioned in Table 1. Most of the studies (six) provided a cohort design, and two of these used cross-sectional study designs. Three of the studies were performed in China, and one each carried out in Italy, Spain, The Netherlands, England, and Europe (the United Kingdom, France, Spain, Lithuania, Norway, and Greece). Six of the studies investigated the impacts of PM_2.5_ exposure on BMI. The studies used a variety of methods to measure exposure, including modeling (Land-use Regression Model (LUR)), GIS platforms, and directed acyclic graphs (DAG), while only one study used satellite remote sensing data. All studies were rated “excellent” according to the NOS standards (>7 stars), as listed in Table 2 and Table 3.

### 3.2. Results of the Meta-Analysis

**Figure 2:**
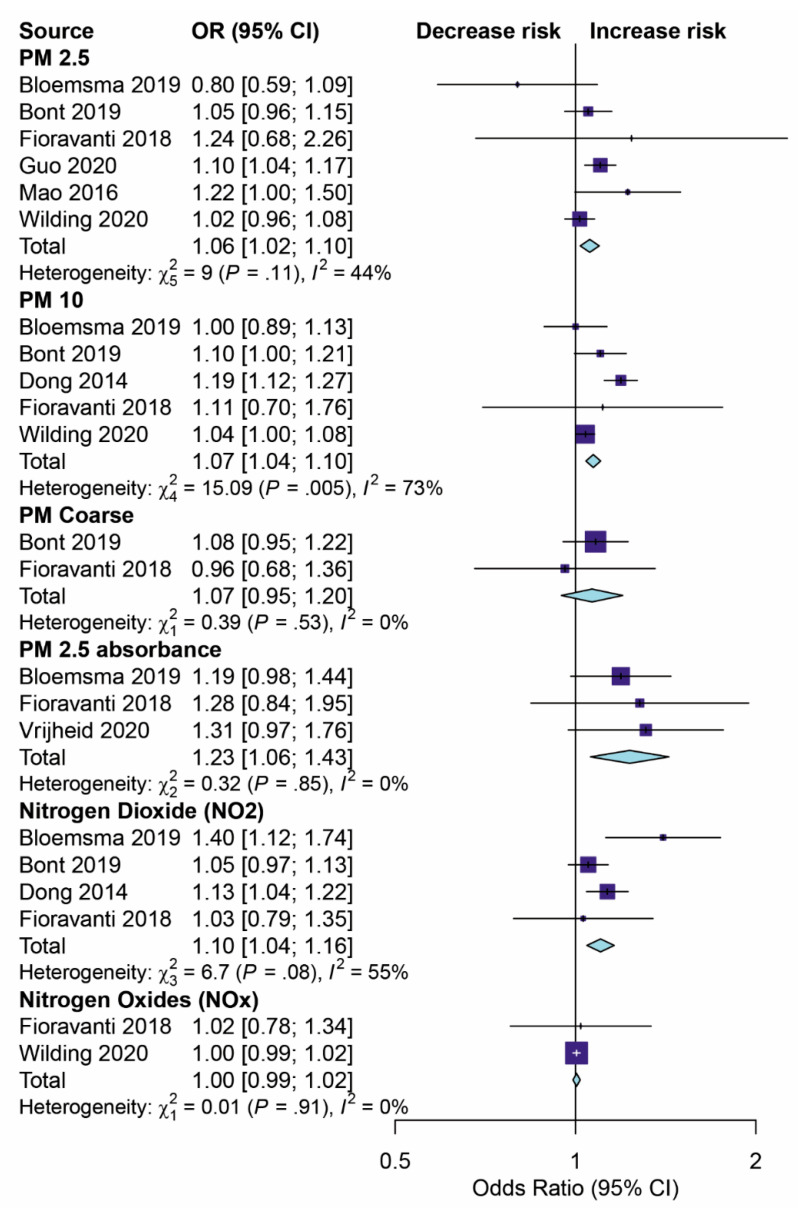
The associations among PM_2.5_, PM_10_, PM_coarse_, PM_absorbance_, NO_2_ and NO_x_ exposure, and obesity in children were assessed by meta-analysis. A Forest plot of the association between exposure to PM_10_ and obesity in children was constructed. The size of the black square corresponding to each study is proportional to the sample size, and the center of each square represents the Effect Size (ES). The horizontal line shows the corresponding 95% CI of the ES. The pooled ES is represented by a hollow diamond. CI, confidence interval; I_2_ = percentage of the total variability due to between-pollution heterogeneity.

The results of the pooled OR model for the eight studies investigating the impact of air pollution on childhood obesity using the meta-analysis evaluation are shown in Figure 2.

PM_2.5_: Six studies [11,18,26,27,28,29] estimated the effect of PM_2.5_ exposure on children’s BMI. The overall effect of the pooled ORs demonstrated a significantly increased risk of childhood weight gain (by 6%) through being exposed to PM_2.5_ (OR 1.06, 95% CI 1.02–1.10, *p* < 0.001) with a median level of heterogeneity (I2 = 44%).

PM_10_: Five studies [25,26,27,28,29] assessed the effects of PM_10_ exposure on BMI in childhood. The summary effect of the pooled ORs was 1.07 (95% CI 1.04–1.10, *p* < 0.001) with high evidence of statistical heterogeneity (I2 = 73%).

PM_coarse_: The studies presented in [26,27] showed that PM_coarse_ exposure was not significantly associated with weight gain or BMI in children. The effect of the pooled ORs was 1.07 (95% CI 0.95–1.20, *p* = 0.291).

PM_2.5absorbance_: Three studies [26,28,30] estimated the association between PM_2.5absorbance_ and children’s BMI. The overall effect of pooled ORs showed a significantly increased risk (by 23%) for weight gain in childhood through being exposed to PM_2.5absorbance_ (OR 1.23, 95% CI 1.06–1.43, *p* < 0.001) with a low level of heterogeneity (I2 = 0%).

NO_2_: Four studies [25,26,27,28] assessed the effect of exposure to NO_2_ on children’s weight. Overall, a significant association between NO_2_ exposure and childhood BMI (OR 1.10, 95% CI 1.04–1.16, *p* < 0.001) was demonstrated with a median level of heterogeneity (I2 = 55%).

NO_x_: Only two studies [26,29] assessed the effect of NO_x_ on children’s weight gain. In summary, the studies showed that exposure to NO_x_ was not significantly associated with childhood BMI (OR 1.00, 95% CI 0.99–1.02, *p* = 0.571) with a low level of heterogeneity (I2 = 0%).

### 3.3. Sensitivity and Subgroup Analysis

Table 4 illustrates the results of the sensitivity and subgroup analyses. The results of the subgroup analysis were stratified according to the area of exposure (Asia and Europe) and the follow-up period (more than 4 years and less than 4 years), which confirmed the robustness of the overall estimates.

For the risk of weight gain or obesity in children from PM_2.5_ exposure, the ORs for the random-effect model in the different areas of exposure, Asia and Europe, were 1.11 (95% CI, 1.04–1.17, *p* < 0.001) and 1.02 (95% CI, 0.97–1.07, *p* < 0.001), respectively. The OR for the random-effects model in follow-up periods of exposure of less than 4 years was 10.3 (95% CI, 0.98–1.08, *p* < 0.001), while the OR for the follow-up periods of exposure that were more than 4 years was 1.08 (95% CI, 1.02–1.14, *p* < 0.001).

The OR for the random-effects model of the association between PM_10_ exposure and weight gain or obesity in the Europe region was 1.04 (95% CI, 1.01–1.07, *p* < 0.001). The equivalent figure for Asia could not be determined because no studies estimated the effects of PM_10_ in Asia. The OR for the random-effects model in follow-up periods of exposure of less than 4 years was 1.07 (95% CI, 1.04–1.10, *p* < 0.001), while the OR for follow-up periods of exposure of more than 4 years was 1.01 (95% CI, 0.89–1.12, *p* < 0.001).

When the effects of pooled ORs for the random-effect model in the Europe region were analyzed, no significant association was found between PM_coarse_ and childhood weight gain (1.07; 95% CI, 0.95–1.20, *p* = 0.291). The figures for the follow-up period were 1.08 (95% CI, 0.95–1.22, *p* = 0.228) for follow-up periods of less than 4 years and 0.96 (95% CI, 0.68–1.36, *p* < 0.817) for follow-up periods of more than 4 years.

The OR for the random-effect model in the Europe region was 1.23 (95% CI, 1.06–1.433, *p* = 0.007), while no studies estimated the effect of PM_2.5absorbance_ in Asia.

The figures for the follow-up period were 1.31 (95% CI, 0.97–1.77, *p* = 0.076) for follow-up periods of less than 4 years and 1.21 (95% CI, 1.01–1.44, *p* = 0.036) for follow-up periods of more than 4 years.

The studies conducted in both Asia and Europe showed a significant effect of NO_2_ on childhood weight gain. The pooled OR from the random-effects model was 1.13 (95% CI, 1.04–1.23, *p* = 0.003) for Asia and 1.08 (95% CI, 1.01–1.16, *p* = 0.032) for Europe. The effects of pooled ORs were 1.09 (95% CI, 1.03–1.15, *p* = 0.014) for follow-up periods of less than 4 years and 1.24 (95% CI, 1.04–1.47, *p* = 0.003) for follow-up periods of more than 4 years.

The OR for the random-effect model of the association between NO_x_ exposure and weight gain or obesity in the Europe region was 1.00 (95% CI, 0.99–1.01, *p* < 0.423), while there were no estimates for the effect of NO_x_ in Asia. The OR was 1.00 (95% CI, 0.99–1.01, *p* = 0.428) for follow-up periods of less than 4 years and 1.02 (95% CI, 0.84–1.24, *p* < 0.839) for follow-up periods of more than 4 years.

### 3.4. Publication Bias of Included Studies

**Figure 3:**
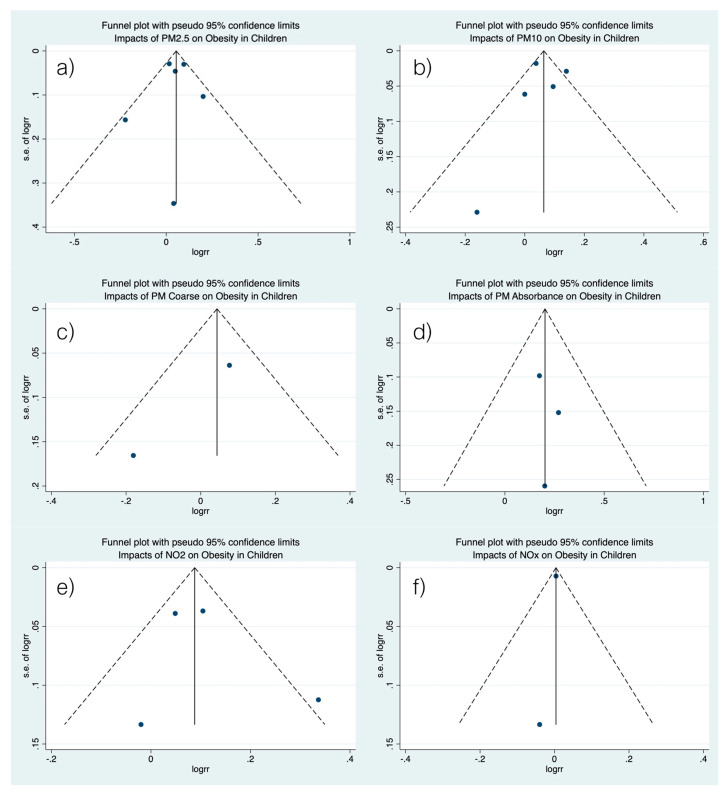
Assessing the publication bias by funnel plot. (**a**) Funnel plot of the association between PM_2.5_ and risk of obesity. (**b**) Funnel plot of the association between PM_10_ and risk of obesity. (**c**) Funnel plot of the association between PM_coarse_ and risk of obesity. (**d**) Funnel plot of the association between PM_2.5absorbrance_ and risk of obesity. (**e**) Funnel plot of the association between NO_2_ and risk of obesity. (**f**) Funnel plot of the association between NO_x_ and risk of obesity.

A statistical study of the bias of publication showed that there was no obvious bias and no statistical difference in publication, as confirmed by the symmetrical funnel plot (Figure 3) and the Begg’s and Egger’s tests shown in Table 5.

## 4. Discussion

The aim of this research was to assess the incidence of obesity in children due to air pollution using a systematic review and meta-analysis. It was found that the air contaminants PM_2.5_, PM_10_, PM_2.5absorbance,_ and NO_2_ play critical roles in childhood obesity. Only a few studies have revealed the effects of NOx and PM_coarse_ on obesity in children. For example, in Fioravanti et al., Bont et al., and Bloemsma et al. [26,27,28], it was indicated that exposure to PM_coarse_ has a tendency to increase childhood BMI by 6%, but no substantial statistical studies have been conducted. At the same time, NO_x_ has rarely been associated with childhood BMI.

The findings of this study, therefore, support the hypothesis that air pollution exposure is correlated with being overweight in children and this leads, in turn, to the development of metabolic disorders [3,31]. Additionally, in the study by Xu et al. [32], it was shown that exposure to PM_2.5_ increases inflammatory responses in adipose tissue resulting from increased weight gain. Meanwhile, in study of An et al. and Toledo-Corral et al. [3,6,31], it was found that air pollution exposure significantly elevates blood glucose and increased the risk of obesity. In addition, air pollution can cause poor sleep, which causes weight gain, according to a study of children in the United States and China [33]. This research found that higher levels of PM dramatically increased the occurrence of sleep problems. Sleep disorders are believed to result in an increase in the body mass index because of reduced leptin levels, elevated thyroid-stimulating hormone levels, increased glucose tolerance, and increased ghrelin levels [34]. Therefore, there may be a link between pollution and weight gain through changes in behavior. Research has shown that people are more likely to stay indoors when there are high levels of air pollution, with increased amounts of time spent sitting and reclining [3,10,35,36]. Due to a decrease in exercise, the amount of energy used for physical activity is reduced, and the risk of obesity increases [3]. Additionally, air pollution leads to health issues that cause people to consume more calories. A study by Xu et al. [32] showed that air pollution increases depression and anxiety while also being associated with increased food intake and insulin resistance.

The physiological mechanisms that cause obesity in children are the result of genetic and environmental interactions [4]. The primary cause is the difference between the consumption of food and nutrition. A rising level of interest has been shown in the connection between environmental exposure and the obesity epidemic [5]. Animal studies have reported that air pollution increases weight gain and leads to metabolic changes [37,38,39]. However, many studies on the association between air pollution and childhood obesity have found that the concentrations of ambient air pollutants, including PM_coarse_, PM_10_, PM_2.5_, and NO_2_, and NO_x_, were associated with increased BMI [25,26,35]. Additionally, previous studies have focused quite extensively on indoor air quality. It is of concern that research has demonstrated that 23% to 35% of school children spend their school days in areas with the highest levels of pollution [40,41].

The duration of exposure, exposure area, and the concentration of air pollution are factors that cause childhood obesity. The period of exposure to PM_2.5_ likely affects the incidence of obesity, but the effect of PM_10_ could not be measured. Specifically, the effects of PM_10_ on the incidence of obesity were not found to be statistically relevant in the subgroup with a follow-up time of > 4 years. The results recommended that PM exposure might cause changes that ultimately contribute to a child becoming overweight or obese. The results, however, tend to be more pronounced for PM_2.5_, possibly due to the comparatively higher biological mechanisms involved [42]. PM_2.5_ could lead to metabolic dysfunction through increased adipose tissue inflammation, oxidative stress, an increased risk of chronic comorbidities, and insufficient physical activity [3,31]. Additionally, some studies have suggested that it may disrupt molecular mechanisms that maintain obesity and affect the occurrence of obesity-related diseases [37,39,43,44]. A study on children reported that it is believed that PM_2.5_ may cause weight gain by causing brain inflammation and raising the risk of developing other chronic diseases, such as cardiovascular diseases, respiratory diseases, and cancer [10]. A recent thorough literature review identified a negative relationship between PM_2.5_ exposure and physical activity versus leisure-time physical inactivity [10,26]. The duration of exposure to air contaminants is also important for determining the impact of air pollution on childhood obesity. On the other hand, no substantial associations between PM_10_ exposure of more than 4 years and obesity have been identified. This may be due to the effect of the exposure area, which is based on exposure to indoor air pollution. For children, the exposure area is mostly schools, as reported by previous studies [40,41]. It was found that children spend about 23–35% of their time at school when the maximum levels of ambient air pollution are observed. In the meantime, PM_2.5absorbance_ could also have an impact on childhood obesity. It is the reflectance at which PM_2.5_ filters have been measured and transformed into absorbance [45]. Although significant combustion sources contribute to PM_2.5absorbance_, ambient concentrations are primarily affected by diesel particles [46]. Composed of elemental carbon, the particles represent incomplete combustion, causing the adsorption of metals and organic compounds, which is tied to cellular effects [47]. As a result, PM_2.5absorbance_ concentrations have been found to be higher at street locations than at urban background sites. Previous studies have reported that traffic congestion is associated with high BMI levels in children aged between 10 and 18 years [13]. The proximity of major streets and time spent in traffic may be the main reasons for the high risk of obesity (23%) identified in this study. Potential mechanisms related to this could be the effect of air pollution on the risks associated with the development of obesity-related metabolic syndrome. Traffic congestion could affect active travel, reduce regular exercise, and probably significantly alter the energy balance, which could increase levels of stress and sleep cycles and, therefore, caloric intake [48]. Another suggested pathway may involve increased oxidative stress and inflammation of adipose tissue as well as decreased muscle glucose uptake [10,26]. Not only particulate matter but also gas-phase species of air pollutants influence the incidence of childhood obesity. In this study, the presence of an average level of NO_2_ increased the risk of obesity by about 9%. NO_2_ is a component of NO_x_ and is an air pollutant measure monitored by the U.S. Environmental Protection Agency (EPA) for compliance. A study by Sutherland et al. [49] found that high average concentrations of ambient NO_2_ occurred in nearby road sections characterized by traffic delays, high traffic volumes, and bus routes. Because of the underlying state of oxidative stress and inflammation that can be attributed to being overweight, it is likely that overweight people are more susceptible to exposure to oxidative and pro-inflammatory compounds such as pollutants [50].

The strengths of this study should be pointed out. The first part of our study involved a comprehensive literature review that was performed by scanning three major databases (PubMed, Science Direct, and Cochrane library). Second, we searched data sources using a systematic search technique without limitations on language, study design, or subject matter. Third, due to the lack of a standard evaluation tool, we evaluated the methodological quality of the included studies and found that most of the included studies met negligible quality standards. The evidence examined is consistent with the quality and quantity of the studies included. Lastly, the meta-analysis used the standard methodology required by the PRISMA checklist. The study used the most recent results and the most suitable statistical methods for analysis.

However, there are a few limitations in this study that need to be pointed out. In previous studies, BMI was based on self-reported information (height and weight) that may be inaccurate and not reliable. To promote accurate and reliable research, studies should be carried out properly with careful regulation. Certain rules must be conformed to when assessing reporting instruments for compliance. Another limitation is that the results of this research are based on epidemiological studies which generally consist of common biases such as the information bias and observer bias. In order to minimize bias in epidemiological studies, exposure and outcome data should be obtained from observational studies or medical records.

## 5. Conclusions

This analysis has shown that increased air pollution is significantly associated with a greater prevalence of overweight or obesity in children. However, studies from developing countries based on a homogenous population subgroup, exposure concentration, medical implications, and a combination of pollutants related to body weight incidence are needed to provide more detailed evidence on the effects of exposure to air pollution at different levels on the body.

## Figures and Tables

**Table 1:** Characteristics of the included studies.

Study (Year, Country)	Study Design	Pollutant (Average Exposure)	Exposure Assessment	Follow-Up (Years)	Subjects (Mean Age: Male, %)	BMI Reference	Adjustment
Dong G.H., et al. (2014, China) [25]	Prospective cohort study	PM_10_: 124.2 μg/m^3^ NO_2_: 19.5 ppb SO_2_: 17.6 ppb O_3_: 27.4 ppb	Followed by the State Environmental protection Administration of China (1992). The daily average concentration of air pollution was calculated based on data from days for which at least 75% valid 1-h values were available from each monitor.	3	8.4 years, 50.4%	BMI (The Centers for Disease Control and Prevention (CDC) BMI growth charts)	Did not adjust for any specific factors
Mao G. et al. (2017, China) [11]	Prospective cohort study	PM_2.5_: 1st trimester: 3.49 mg^2^m^3^ 2nd trimester: 3.42 mg^2^m^3^ 3rd trimester: 3.64 mg^2^m^3^ Whole pregnancy: 3.17 mg^2^m^3^ First 2 years of life: 3.01 mg^2^m^3^	The PROC GEOCODE procedure of SAS 9.4 (SAS Institute Inc.) was used and matched to the nearest monitor using ArcGIS 10.2 (Esri).	3	6.7 years, 41.36%	BMI the SAS Program for the 2000 CDC Growth Charts provided by the Centers for Disease Control and Prevention (CDC) 2011.	Maternal age at delivery, race/ethnicity, education level, smoking status during pregnancy, diabetes, marital status, household income per year, maternal prepregnancy body mass index (MPBMI), season of delivery, preterm birth, birth weight, and breastfeeding
Fioravanti S. et al. (2018, Italy) [26]	Cohort study	PM_2.5_: 5 μg/m^3^ PM_10_: 10 μg/m^3^ PM_coarse_: 5 μg/m^3^ PM_absorbance_: 10 μg/m^3^ NO_x_: 20 μg/m^3^ NO_2_: 10 μg/m^3^	Land-use regression models (LUR)	4–8	50 months, 50.6%	BMI (WHO Growth reference 2007)	Maternal educational level, paternal educational level, maternal pre-pregnancy BMI, maternal smoking during pregnancy, maternal age at delivery, gestational age, child’s birthweight, breastfeeding duration, and age at weaning.
Bont J.D. et al. (2019, Spain) [27]	Cross-sectional study	PM_2.5_: 2.7 μg/m^3^ PM_10_: 5.6 μg/m^3^ PM_coarse_: 3.7 μg/m^3^ NO_2_: 13.7 μg/m^3^	Land-use regression models (LUR)	0.5	8.4 years, 57%	BMI (WHO Growth reference 2007)	Parental education, employment status, and country of birth, maternal smoking during pregnancy, child’s adoption status, exposure to environmental tobacco smoke (ETS) at home, number of siblings, and physical activity
Bloemsma L.D. et al. (2019, the Netherlands) [28]	Cohort study	PM_2.5_: 1.17 μg/m^3^ PM_2.5absorbance_: 2.5 × 10^−5^ μg/m^3^ PM_10_: 1.06 μg/m^3^ NO_2_: 8.9 μg/m^3^	Land-use regression models (LUR)	17	The youngest 2.5–3.5 and the oldest 16–19, 51.9%	BMI (International Obesity Task Force cutoffs)	Age, sex, maternal and paternal levels of education, maternal smoking during pregnancy, parental smoking in the child’s home and neighborhood, SES, and region
Guo Q. et al. (2020, China) [18]	Cross sectional study	PM_2.5_: 59.8 μg/m^3^	A machine-learning model with satellite remote sensing measurements and historical emission inventories as input	5	6–18 years, 48.29%	BMI (The Chinese national standard Screening for Overweight and Obesity among School-age Children and Adolescents	(1) Sociodemographic factors, including gender, age category, urbanity, region, economic level, day or boarding school, and educational level and occupation of mother; (2) dietary intake, including total water intake, food intake, and beverages intake; (3) time-activity patterns; and (4) indicators of indoor air pollution, including household cooking fuel type, household heating fuel type, school heating fuel type, home ventilation time, and secondhand smoke duration
Wilding S. et al. (2020, England) [29]	Cross sectional study	PM_2.5_: 13.1 μg/m^3^ PM_10_: 18.6 μg/m^3^ NO_x_: 40.2 μg/m^3^	Average level of background air pollution modelled on an annual basis. The annual mean for each metric is provided for 1 km grids across the United Kingdom. A spatially weighted average for each Lower and Middle layer Super Output Areas for PM_5_, PM_10_, and NO_x_ was calculated based on levels of background air pollution modelled by the UK Department for Environment Food and Rural Affairs	-	7–11 years, did not report	BMI (The National Obesity Observatory and population cut-off used in NCMP reports.	Did not adjust for any specific factors
Vrijheid M. et al. (2020, the United Kingdom, France, Spain, Lithuania, Norway, and Greece) [30]	Cohort study	Outdoor PM_2.5absorbance_: 0.41 × 10^−5^/m^−1^ Indoor PM_2.5absorbance_: 0.50 × 10^−5^/m^−1^ Indoor NO_2_: 92.8 μg/m^3^	Land-use regression models (LUR)	6–9	8.1 years, 54.7%	BMI (WHO Growth reference 2007)	Sex, maternal BMI, maternal education, maternal age at conception, parity, and prenatal country of origin.

**Table 2:** Assessment of quality using the Newcastle-Ottawa quality assessment scale (Cohort study).

Study	Selection				Comparability	Outcome			Score
	1	2	3	4	1	1	2	3	
Dong, et al. (2014) [25]	*	*	*	*	**	*	*	*	9
Mao, et al. (2017) [11]	*	*	*	*	**	*	*	*	9
Fioravanti, et. al. (2018) [26]	*	*	*	*	**	*	*	*	9
Bloemsma, et al. (2019) [28]	*	*	*	*	**	*	*	*	9
Vrijheid, et al. (2020) [30]	*	*	*	*	**	*	*	*	9

Note: the symbol “*” indicates that the corresponding item is applicable to the study, so the corresponding score is assigned to the corresponding study with a maximum of two stars (“**”) that can be given for comparability. Score is Newcastle-Ottawa Quality Assessment Scale.

**Table 3:** Assessment of quality using the Newcastle-Ottawa quality assessment scale (Cross sectional study).

Study	Selection				Comparability	Outcome		Score
	1	2	3	4	1	1	2	
Bont, et al. (2019) [27]	*	*	*	*	**	*	*	8
Guo, et al. (2020) [18]	*	*	*	*	**	*	*	8
Wilding, et al. (2020) [29]	*	*	*	*	**	*	*	8

Note: the symbol “*” indicates that the corresponding item is applicable to the study, so the corresponding score is assigned to the corresponding study with a maximum of two stars (“**”) that can be given for comparability. Score is Newcastle-Ottawa Quality Assessment Scale.

**Table 4:** Sensitivity and subgroup analyses.

Characteristic		Model		Area of Exposure		Period of Follow-Up	
		Fixed-Effect Model	Random-Effects Model	Asia	Europe	Less than 4 Years	More than 4 Years
**PM_2.5_**							
**Adjusted OR (95% CI)**		1.05 (1.01–1.09)	1.05 (1.01–1.09)	1.11 (1.04–1.17)	1.02 (0.97–1.07)	1.03 (0.98–1.08)	1.08 (1.02–1.14)
**Heterogeneity**	***I*^2^ value (%)**	47.2	47.2	0.0	18.2	24.5	62.5
	***p*-value**	<0.001	<0.001	<0.001	<0.001	<0.001	<0.001
**PM_10_**							
**Adjusted OR (95% CI)**		1.06 (1.04–1.10)	1.07 (1.04–1.10)	N/A	1.04 (1.01–1.07)	1.07 (1.04–1.10)	1.01 (0.89–1.12)
**Heterogeneity**	***I*^2^ value (%)**	71.9	71.9	N/A	0.0	84.6	0.0
	***p*-value**	<0.001	<0.001	N/A	<0.001	<0.001	<0.001
**PM_coarse_**							
**Adjusted OR (95% CI)**		1.06 (0.94–1.19)	1.07 (0.95–1.20)	N/A	1.07 (0.95–1.20)	1.08 (0.95–1.22)	0.96 (0.68–1.36)
**Heterogeneity**	***I*^2^ value (%)**	0.0	0.0	N/A	0.0	–	–
	***p*-value**	<0.001	0.291	N/A	0.291	0.228	0.817
**PM_2.5abs_**							
**Adjusted OR (95% CI)**		1.22 (1.04–1.41)	1.23 (1.06–1.43)	N/A	1.23 (1.06–1.43)	1.31 (0.97–1.77)	1.21 (1.01–1.44)
**Heterogeneity**	***I*^2^ value (%)**	0.0	0.0	N/A	0.0	–	0.0
	***p*-value**	<0.001	0.007	N/A	0.007	0.076	0.036
**NO_2_**							
**Adjusted OR (95% CI)**		1.09 (1.04–1.15)	1.10 (1.04–1.16)	1.13 (1.04–1.23)	1.08 (1.01–1.16)	1.09 (1.03–1.15)	1.24 (1.04–1.47)
**Heterogeneity**	***I*^2^ value (%)**	47.6	55.2	–	66.5	41.1	66.8
	***p*-value**	<0.001	<0.001	0.003	0.032	0.014	0.003
**NO_x_**							
**Adjusted OR (95% CI)**		1.00 (0.99–1.02)	1.00 (0.99–1.02)	N/A	1.00 (0.99–1.01)	1.00 (0.99–1.01)	1.02 (0.84–1.24)
**Heterogeneity**	***I*^2^ value (%)**	0.0	0.0	N/A	0.0	0.0	0.0
	***p*-value**	<0.001	0.571	N/A	0.423	0.428	0.839

**Table 5:** Begg’s test and Egger’s test of publication bias.

Pollutant	Begg’s Test		Egger’s Test						
	Z	*p*-Value	Std_Eff	Coef.	Std. Err.	*t*	*p* > ltl	[95% Conf. Interval]	*p*-Value
PM_2.5_	0.00	1.00	slope	0.062	0.048	1.3	0.262	−0.07 to 0.19	0.839
			bias	−0.023	1.05	−0.22	0.84	−3.14 to 2.69	
PM_10_	−0.024	1.00	slope	0.68	0.49	1.38	0.26	−0.88 to 0.22	0.929
			bias	−0.15	1.54	−0.10	0.93	−5.07 to 4.78	
PM_coarse_	0.00	1.00	slope	0.24	–	–	–	–	–
			bias	−2.53	–	–	–	–	
PM_2.5absorbance_	0.00	1.00	slope	0.15	0.10	1.44	0.39	−1.17 to 1.47	0.684
			bias	0.41	0.76	0.54	0.68	−9.28 to 10.11	
NO_2_	0.34	0.73	slope	0.04	0.09	0.47	0.69	−0.36 to 0.45	0.651
			bias	0.97	1.84	0.53	0.651	−6.96 to 8.91	
NOx	0.00	1.00	slope	0.01	–	–	–	–	–

## Data Availability

The data presented in this study are available in Effect of Air Pollution on Obesity in Children: A Systematic Review and Meta-Analysis.
